# Glanders and Its Suppression

**Published:** 1899-02

**Authors:** J. M. Wright

**Affiliations:** Professor of Pathology and Contagious Diseases, M’killip Veterinary College; Assistant State Veterinarian of Illinois


### GLANDERS AND ITS SUPPRESSION; EXPERIMENTS WITH MALLEIN.[1]

By J. M. Wright, M.D.C.,
PROFESSOR OF PATHOLOGY AND CONTAGIOUS DISEASES, M'KILLIP VETERINARY COLLEGE; ASSISTANT STATE VETERINARIAN OF ILLINOIS.

(Concluded from page 16.)

Mallein was first introduced by Kalning, Preusse, and Pearson, by making a culture of the bacilli of glanders in the proper culture-media. I consider it of great value as an assisting agent in making diagnosis in occult cases. In fact, I have never known it to fail in the work for which it is intended. I have used it on more than 2000 horses which were diseased or had been exposed. I have used mallein experimentally to test its value as a diagnostic agent on 138 healthy horses, of all ages, at all seasons of the year; on 6 horses with well-marked generalized melanosis, 13 cases of pleurisy, 16 cases of pneumonia, 3 cases of pleuro-pneumonia, 24 cases of influenza, 3 cases of purpura hemorrhagica, and 14 cases of lymphangitis, two of which were in a state of suppuration—*i. e.*, having numerous little abscesses from the hoof to the stifle, resembling very much the buttons and ulcers of farcy. None of these animals showed the slightest degree of reaction, either by causing an attenuation of temperature or any systemic disturbance. The use of mallein as an agent in ascertaining the animals which contain the bacilli cannot be extolled too highly. I would recommend its use in all doubtful cases, and on all horses, mules, or asses that have been exposed to glanders.

To use mallein intelligently, one must, in the first place, be sure that he has a good quality of the agent, and in making the test I would recommend the following method: The first mallein-test should not be given earlier than ten days after the last glandered animal has been destroyed, nor later than three weeks, and the premises in the meantime should be thoroughly cleaned and disinfected. I have observed, if the test be given immediately after the last glandered horse has been destroyed, that some will not react, but will do so later at a second test. Such a thing will rarely occur if two weeks are allowed to elapse after the last glandered animal is destroyed before giving the test. The test should cover a period of two days, and the first day the temperature of the animals should be taken at 6 A.M., and again at 12 M., and again at 6 P.M., to ascertain their normal temperatures. At

9 P.M., proceed to give the injection with a hypodermic syringe in the following manner: First, thoroughly sterilize the skin on the cervical region beneath the mane, with a strong antiseptic; then burn the hypodermic needle in the flame of an alcohol-lamp, load it with 2½ c.c. of mallein, and inject beneath the skin at the point indicated in the cervical region. Great care should be exercised in using the hypodermic needle; it should be used but for one animal, or be thoroughly sterilized in the flame of an alcohol-lamp before each injection, as neglect in this particular may result in the transmission of the disease from one animal to another. On the second day the temperature should be taken at 6 and 9 A.M., 12 M., and 3, 6, and 9 P.M., which completes the test.

Great care should be exercised in taking the temperature from 6 A.M. to 9 P.M., for the simple reason that some of the animals may not react until late in the day. I have in one instance observed an animal that did not show the slightest disturbance until twenty-three and a half hours had passed after the injection of the mallein, and at the expiration of that time he commenced to react to an extreme degree, and the disturbance was so great that he died at the end of the fourth day. Postmortem showed the glanderous condition to be far advanced in the lungs and the liver badly affected. In a number of instances I have observed that the animals have not shown any indication of reaction until eighteen, twenty, twenty-one, and twenty-two hours have passed, while all experimenters know the reaction will usually be manifest at a period ranging from eight to fifteen hours.

All animals that show an elevation of temperature of four or more degrees, have a large and painful swelling at the point of injection, excessive systemic disturbance, loss of appetite and depression, should be destroyed at once. All animals showing an elevation of temperature of two or more degrees, with a moderate amount of swelling at the point of injection, no noticeable amount of depression, no loss of appetite, no systemic disturbance, except the elevation of temperature, should be isolated and kept from all other horses, under quarantine, for from three to six months, and inspected once each week by a competent veterinary surgeon, and they should be out in the open air and sunshine, with moderate work, but never enough to produce exhaustion.

At the end of this period they should be given another mallein-test, and all animals showing reaction at the last test should be destroyed, and those which do not should be released from quarantine as healthy.

It is claimed by some very eminent men that mallein will cure occult glanders. Is it not possible mallein is given credit for doing what nature has done? Let us look at the question for a moment. As I have already said, an average of 40 per cent. of all animals exposed are infected with the virus of glanders. If these be kept at moderate work, out in the open air and sunshine, and be housed in pure, clean, well-ventilated barns, with plenty of good, pure water and nourishing food, less than 5 per cent. of the 40 per cent. will develop into clinical glanders, but will become free of the infection. In such cases if mallein were used it would be given the credit.

I have been unable, by the use of mallein as a therapeutical agent, to determine the percentage of developments into clinical cases, or to diminish the number of mortalities, although I have noticed emaciated animals, after its use, apparently improve in flesh, and occasionally they will continue to improve for three, four, and five months, and then develop into clinical cases of glanders. I have given doses every seven days, but found that after four or five doses there would be no reaction, as far as elevation of temperature is concerned, but a part of them would develop into clinical cases. I have tried to accomplish the desired end by giving a dose every thirty days, and with others every sixty days, but the results were negative, and some of them would develop into clinical cases. In all these animals the treatment was the same as that given those which slightly reacted from the test. Taking all these facts into consideration, I do not feel justified in recommending the use of mallein as a curative agent, except experimentally, fearing that inexperienced persons may release animals from quarantine that should be held.

Before beginning the fight with this enemy it will be well for us to have some knowledge of its power of resistance. First, we find the true cause of this disease is a bacillus, slightly shorter and decidedly thicker than the bacillus of tuberculosis, rounded at the ends and slightly curved upon itself. It can be cultivated in various culture-media. Outside the body it will develop in the proper media at a temperature above 68° F. and below 113° F. At a temperature above 113° F. or below 68° F. its growth is arrested and it soon perishes. It is destroyed in a temperature of 145° F. in ten minutes and in a temperature of 176° F. in five minutes. If it should be in water it will resist the excessive heat longer than in a dry state. A 3 per cent. solution of carbolic acid will destroy it in five minutes, a 1 per cent. solution of permanganate of potash in two minutes, and 1 to 5000 bichloride of mercury in two minutes. It is destroyed by desiccation in one week. It will live in water above 80° F. from fifteen to twenty days. It will resist putrefaction from fifteen to twenty-four days. It will live in distilled water six days. A low temperature will destroy it very quickly.

After the removal or destruction of the last animal affected by the disease I would recommend removal and burning of all loose material, such as refuse, hay, bedding, etc. Water- and feed-troughs and stalls of the stable should be thoroughly scrubbed with a broom and boiling water for the purpose of thoroughly destroying all materials which may have accumulated and dried there. To make the cleansing more complete I would advise rescrubbing with 1 per cent. solution of permanganate of potash, 1 to 5000 solution of bichloride of mercury, or 3 per cent. solution of carbolic acid, after which whitewash all the above-mentioned places. The wagon-tongue and neck-yoke should be scrubbed and washed with one of these solutions. All harness, bridles, bridle-bits, etc., should be thoroughly washed in a solution of permanganate of potash and oiled.

A field, pasture, stream of water, stable, or barn will become perfectly free from the bacillus in summer-time in less than ninety days, without any treatment, and in a week or less in fall or winter, the time varying according to variations in temperature. It is absolutely unnecessary to hold any premises under quarantine, where the animals have been removed and where it is impracticable to properly disinfect them, for a longer period.

